# An age and space structured SIR model describing the Covid-19 pandemic

**DOI:** 10.1186/s13362-020-00090-4

**Published:** 2020-08-08

**Authors:** Rinaldo M. Colombo, Mauro Garavello, Francesca Marcellini, Elena Rossi

**Affiliations:** 1 INdaM Unit, University of Brescia, Via Branze 38, Brescia, 25123 Italy; 2 Dept. of Mathematics and its Applications, University of Milano-Bicocca, Via R. Cozzi 55, Milan, 20125 Italy

**Keywords:** Age and Space Structured SIR Model, Differential Equations in Epidemic Modeling, Covid-19 Modeling

## Abstract

We present an epidemic model capable of describing key features of the Covid-19 pandemic. While capturing several qualitative properties of the virus spreading, it allows to compute the basic reproduction number, the number of deaths due to the virus and various other statistics. Numerical integrations are used to illustrate the adherence of the evolutions described by the model to specific well known real features of the present pandemic. In particular, this model is consistent with the well known relevance of quarantine, shows the dramatic role of care houses and accounts for the increase in the death toll when spatial movements are not constrained.

**Electronic Supplementary Material:**

The online version of this article (10.1186/s13362-020-00090-4) contains supplementary material.

## Introduction

Our aim here is to present a model that contains key features of the Covid-19 outbreak. It uses as starting point the classical SIR class of models, see [1, Sect. 13.5] but is thoroughly adapted to the present day pandemic. Indeed, its key features are:

- Infected individuals are distinguished between Infective (*I*) and Hospitalized (*H*). The former ones do spread the disease, while the latter ones, hospitalized or in quarantine, don’t. We thus consider the four populations of Susceptible (*S*), Infective (*I*), Hospitalized (*H*) and Recovered (*R*) individuals.
- The four densities *S*, *I*, *H*, *R* depend on time $t \in {\mathbb {R}}_{+}$, on age $a \in {\mathbb {R}}_{+}$ and on a space coordinate $x \in {\mathbb {R}}^{2}$. $S (t,a,x)$ (respectively $I (t,a,x)$, $H (t,a,x)$, $R (t,a,x)$) quantifies the individuals of type *S* (respectively *I*, *H*, *R*) that at time *t* are of age *a* and are sited at position *x*.
- Infection is propagated in space: *S* individuals can be infected by *I* individuals of all ages, provided they are at the same time at a distance less than a given threshold. *H* individuals do not infect anyone.
- *S*, *I* and *R* individuals move in the space domain with a time, age and space dependent velocity. *H* individuals are not assumed to move. A further distinction of *S* (respectively *I* and *R*) individuals according to their different destinations is also possible, through further distinction into subclasses of the various populations.
- At a given time, age and space dependent rate, infective individuals are hospitalized or constrained to quarantine, thus entering the *H* population. Both infective and hospitalized individuals recover or die at time, age and space dependent rates.

**Figure 1:**
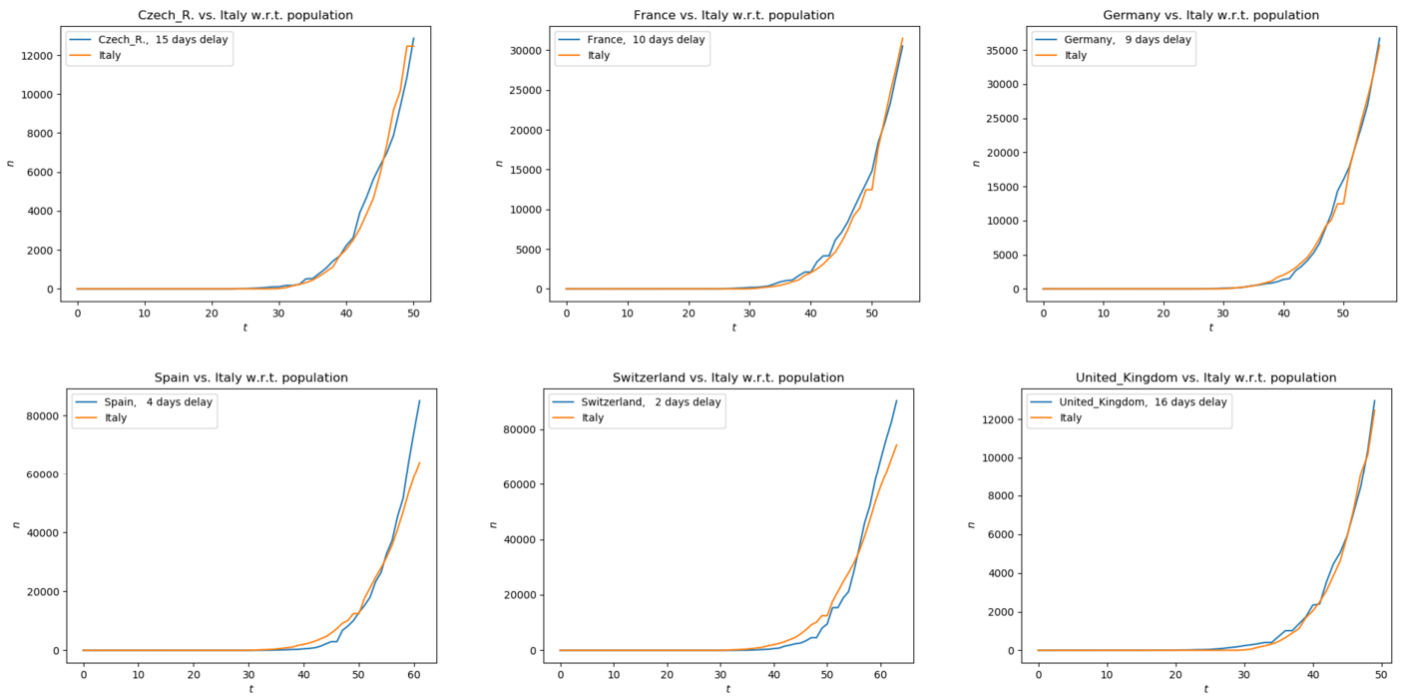
Number of confirmed Covid-19 cases in different European countries, scaled to the Italian population, as a function of time. Along each horizontal axis, time is measured in days starting January 22nd, 2020, each country compared to Italy being delayed by the amount indicated in each top left corner. The numbers of cases are taken from [2], the populations’ data from [3]

Before passing to the rigorous description of the model, we recall that different countries reacted to Covid-19 in different ways. Nevertheless, the initial stages of the virus spreading are quantitatively quite similar in the different countries, once they are correctly scaled with respect to the overall population: their only difference is essentially a time delay, see Fig. 1. These striking similarities definitely justify the search for a unique model able to describe the initial virus spreading.

As is well known, different countries are taking different measures to contrast the pandemic. Key differences typically concern the strength of *lock down*, constraining individuals’ movements and contacts at different levels. In the model presented below, these differences can be covered through *ad hoc* choices of a function, namely *ρ*, that can describe various types of contagion. We describe below the effects of different quarantine policies, from the pandemic evolution point of view. As a further example, we show some effects of care houses, that is of places where the virus spreads faster, in accelerating the infection, also in the case where only one age class is present in the care house. A further feature specific to the present model, is the spatial structure. With a numerical integration we show that people’s movements may well speed up the pandemic. In this connection, we note the possible interest in further extending the model presented below to possibly cover also some of the consequences of the pandemic at the economic/industrial/financial levels.

Below, we use the model here introduced to describe qualitatively relevant features of the Covid-19 pandemic. At a quantitative level, the use of the present model relies on the availability of reliable data, which is not always possible. In this connection, we refer for instance to [4] for the description of a method able to cope with uncertain data.

We stress our interest in providing *qualitative* information, able to compare various strategies to contrast the infection. We present a realistic framework comprising all those features that are presently undoubtedly relevant in the pandemic diffusion: age, movements, quarantine, infection *“at a distance”*, …

For completeness, we refer to [5] for a different approach to the modeling of the Covid-19 pandemic, also based on integro–differential equations. Differently from the model therein, here we do not resort to delayed terms, partly using the standard coupling between the different equations describing the passage between different populations. A related work considering an epidemic model with age structure and immigration is described in [6].

The next section is devoted to a rigorous formulation of the model, of its key approximations and of its main properties. Then, by means of numerical integrations, we show key qualitative properties of the solutions, which agree with well known properties of the Covid-19 pandemic. In these integrations, the various functions entering the model definitions are chosen in agreement with publicly available data.

## The model

A population lives in a region $\mathcal{X} \subseteq {\mathbb {R}}^{n}$ and is subject to an infective disease. Clearly, we typically set $n=2$, but also the case $n=1$ can be of use in a simplified framework.

Throughout, $S = S (t,a,x)$ is the number of susceptible individuals at time $t \in {\mathbb {R}}_{+}$, of age $a \in {\mathbb {R}}_{+}$ at position $x \in\mathcal{X}$. When infected, susceptible individuals enter the *I* population, i.e., they first turn into being infected and infective, possibly asymptomatic. These individuals are then hospitalized or set into quarantine at a rate $\kappa= \kappa(t,a,x)$ and, when this happens, we label them as $H = H (t,a,x)$. Both *I*, respectively *H*, individuals may possibly recover at rates $\vartheta = \vartheta (t,a,x)$, respectively $\eta= \eta(t,a,x)$, entering the population labeled as $R = R (t,a,x)$. We keep the *R* population distinct from the *S* one, assuming that those who recover are immune to any further infection. A different assumption, namely that those who recover are not immune, amounts, for instance, to add further terms coupling the last equation to the previous ones in (1).

*S* (respectively *I* and *R*) individuals move in space with the assigned velocity $v_{S} = v_{S} (t,a,x)$ (respectively $v_{I} = v_{I} (t,a,x)$ and $v_{R} = v_{R} (t,a,x)$). Depending on the geographical scale at which the present model is applied, it might be of use to exploit crowd dynamics models, see for instance [7]. At a different scale, $v_{S}, v_{I}$ and $v_{R}$ may also describe the collective movements of relatively large sets of individuals heading towards regions less hit by the pandemic.

Independently of the movements’ scale, when individuals of the same type, say *S*, follow different routes, we distinguish *S* into different components, say $S^{1}, S^{2}, \ldots$ , and we assign them the different velocities $v_{S^{1}}, v_{S^{2}}, \ldots$ , following a usual approach in crowd dynamics, see for instance [8]. However, this latter distinction introduces a non trivial formal complexity, with no relevance at the level of the present initial description and we leave the corresponding technical details to a later work.

The disease is transmitted by *I* individuals to *S* ones that are, at any given time, geographically close, independently of age.

We are thus lead to the model

1 $$ \textstyle\begin{cases} \partial_{t} S + \partial_{a} S + \mathop {\operatorname {div}}_{x} (v_{S} S) + \mu_{S} S = - (\rho\otimes I) S, \\ \partial_{t} I + \partial_{a} I + \mathop {\operatorname {div}}_{x} (v_{I} I) + \mu_{I} I = (\rho\otimes I) S - \kappa I - \vartheta I, \\ \partial_{t} H + \partial_{a} H + \mu_{H} H = + \kappa I - \eta H, \\ \partial_{t} R + \partial_{a} R + \mathop {\operatorname {div}}_{x} (v_{R} R) + \mu_{R} R = + \vartheta I + \eta H. \end{cases} $$

Here, the term *κI* describes the speed at which *I* individuals are hospitalized or put into quarantine. Similarly, the term *ϑI* is the speed at which *I* individuals recover, while hospitalized individuals recover with a rate *ηH*. As usual, for $A = S,I,H,R$, $\mu_{A}$ is the mortality of the individuals of type *A*. All the above parameters, in particular the mortality rates, are time, age and space dependent.

In (1), for merely typographical reasons, we use the abbreviation

2 $$ (\rho\otimes I) (t,a,x) = \int_{{\mathbb {R}}_{+}} \int_{\mathcal{X}} \rho(t, a, \alpha, x, \xi) I (t,\alpha, \xi) \, \mathrm {d}{\xi} \,\mathrm {d}{\alpha}. $$

This key term is the rate at which susceptible individuals get infected. The function *ρ* plays a fundamental role, for it describes the dynamics of the disease transmission. Various properties of the function *ρ* have a clear counterpart on the real characteristics of the virus spreading. Depending on the particular scenario that is under consideration, different choices of *ρ* are due. However, the following key property is essential:

### Virus transmission

Assume that *δ* is the smallest distance satisfying

3 $$ { \Vert x - \xi \Vert } > \delta\quad\Longrightarrow\quad\rho(t, a, \alpha, x, \xi) = 0, $$

*x* and *ξ* being positions in $\mathcal{X}$. Then, *δ* represents the maximal distance at which the virus can be transmitted. Clearly, the speed of the infection is infinite, within the distance *δ*. Note that it is very reasonable to consider also situations where the above bound *δ* is age and/or space dependent. Indeed, this allows the specific consideration of environments where individuals of different ages have different behaviors, such as schools.

Note that a careful choice of the function *ρ* allows the description of the different scales at which the disease can be transmitted. Scaling conveniently the dependence of *ρ* on ${ \Vert x-\xi \Vert }$, (3) allows, for instance, to account both for the more probable infection at low distance and for that, less probable, caused by tiny droplets that can cover relatively high distances.

Moreover, suitable choices of *ρ* may well describe various specific situations. For instance, the dependence of *ρ* on the age variables *a* and *α* allows to consider situations in which contagion is restricted—or more/less prominent—among individuals of specific ages, for instance of the same age.

Model (1) needs to be complemented with initial and boundary data, say

4 $$\begin{aligned} \begin{aligned} &(a,x) \in {\mathbb {R}}_{+} \times\mathcal{X}, \qquad\quad(t,x) \in {\mathbb {R}}_{+} \times\mathcal{X},\qquad\qquad (t,a,\xi) \in {\mathbb {R}}_{+} \times {\mathbb {R}}_{+} \times\partial\mathcal{X} \\ &\textstyle\begin{cases} S (0,a,x) = S_{o} (a,x) \\ I (0,a,x) = I_{o} (a,x) \\ H (0,a,x) = H_{o} (a,x) \\ R (0,a,x) = R_{o} (a,x) \end{cases}\displaystyle \textstyle\begin{cases} S (t,0,x) = S_{b} (t,x), \\ I (t,0,x) = I_{b} (t,x), \\ H (t,0,x) = H_{b} (t,x), \\ R (t,0,x) = R_{b} (t,x), \end{cases}\displaystyle \textstyle\begin{cases} S (t,a,\xi) = S_{\partial}(t,a,\xi), \\ I (t,a,\xi) = I_{\partial}(t,a,\xi), \\ H (t,a,\xi) = H_{\partial}(t,a,\xi), \\ R (t,a,\xi) = R_{\partial}(t,a,\xi), \end{cases}\displaystyle \end{aligned} \end{aligned}$$

which have to be chosen according to the specific situation under study. The only general constraint to be imposed on these data, besides obvious minimal regularity conditions necessary from the analytic point of view, is that newborns, corresponding to $a=0$, are mostly in the *S* population. In other words, while the analytic well posedness is completely independent of this requirement, we expect that in every realistic application we have

5 $$ \forall (t,x) \in {\mathbb {R}}_{+} \times\mathcal{X},\quad I_{b} (t,x) = H_{b} (t,x) = R_{b} (t,x) = 0. $$

As it is well known, in the case of general balance laws, assigning and understanding the role of the boundary condition along the spatial boundary $\partial\mathcal{X}$ requires particular care, see [9–11]. Here, though not strictly necessary form the analytic point of view, we assume the individuals’ velocities to be assigned are time, age and space dependent functions, so that boundary data are essential whenever the velocities point inward $\mathcal{X}$, while they are neglected when velocities point outward.

A relevant time dependent statistics commonly used to quantify the spreading speed of the disease is the basic reproduction number [12, Sect. 10.2], typically denoted by $\mathcal{R}_{o}$:

$$\begin{aligned} \mathcal{R}_{o} (t) = \frac{\text{(average infection rate} \times\text{number of susceptibles at time $t$)}}{\text{(average recovery rate at time $t$)}}. \end{aligned}$$

Above *“average”* refers to both age and space averages. In the present *dynamic* setting, this index needs to be time dependent. Moreover, the presence of 2 different populations of ill individuals, namely the infective (*I*) and the hospitalized (*H*) ones, allows for the introduction of two indexes inspired by $\mathcal{R}_{o}$. The first one, say $\mathcal{R}_{o}$, considers only the infective ones while the latter, say $\mathcal{Q}_{o}$, comprises also the hospitalized ones:

6 $$ \begin{aligned} &\mathcal{R}_{o} (t) = \frac{\iint \iint\rho(t,a,\alpha,x,\xi) I (t,\alpha,\xi) S (t,a,x) \,\mathrm {d}{\alpha} \,\mathrm {d}{\xi} \,\mathrm {d}{a} \,\mathrm {d}{x}}{\iint(\kappa+ \vartheta + \mu_{I})I(t,a,x) \,\mathrm {d}{a} \,\mathrm {d}{x}}, \\ &\mathcal{Q}_{o} (t) = \frac{\iint \iint\rho(t,a,\alpha,x,\xi) I (t,\alpha,\xi) S (t,a,x) \,\mathrm {d}{\alpha} \,\mathrm {d}{\xi} \,\mathrm {d}{a} \,\mathrm {d}{x}}{\iint ( (\vartheta + \mu_{I}) I (t,a,x) + (\eta+ \mu_{H}) H (t,a,x) ) \,\mathrm {d}{a} \,\mathrm {d}{x}}, \end{aligned} $$

where we shortened $\kappa= \kappa(t,a,x)$, $\vartheta = \vartheta (t,a,x)$, $\eta= \eta(t,a,x)$, $\mu_{I} = \mu_{I} (t,a,x)$ and $\mu_{H} = \mu_{H} (t,a,x)$.

The above definitions are justified by the following necessary and sufficient conditions, that hold provided the inflow/outflow in/from $\mathcal{X}$ vanishes and provided no newborn is ill:

$$ \begin{aligned} &\mathcal{R}_{o} (t) \lessgtr1 \quad\iff\quad \frac{d}{d t} \biggl( \iint I (t,a,x) \,\mathrm {d}{a} \,\mathrm {d}{x} \biggr) \lessgtr0 , \\ &\mathcal{Q}_{o} (t) \lessgtr1 \quad\iff\quad \frac{d}{d t} \biggl( \iint \bigl( I (t,a,x) + H (t,a,x) \bigr) \,\mathrm {d}{a} \,\mathrm {d}{x} \biggr) \lessgtr0. \end{aligned} $$

The proofs amount to elementary applications of the Divergence Theorem and, hence, are omitted.

In other words, $\mathcal{R}_{o} (t)$ describes the instantaneous variation of the number of infective (*I*) individuals at time *t*, while $\mathcal{Q}_{o} (t)$ describes that of the total number of ill ($I+H$) individuals. Thus, $\mathcal{R}_{0}$ measures the danger of being infected, while $\mathcal{Q}_{0}$ measures the overall effect of the disease spreading, coherently with (3).

For completeness, we note that the above definitions can be slightly simplified neglecting the mortality terms, obtaining

$$\begin{aligned} &\tilde{\mathcal{R}}_{o} (t) = \frac{\iint \iint\rho(t,a,\alpha,x,\xi) I (t,\alpha,\xi) S (t,a,x) \,\mathrm {d}{\alpha} \,\mathrm {d}{\xi} \,\mathrm {d}{a} \,\mathrm {d}{x}}{\iint (\kappa(t,a,x) + \vartheta (t,a,x) )I(t,a,x) \,\mathrm {d}{a} \,\mathrm {d}{x}}, \\ &\tilde{\mathcal{Q}}_{o} (t) = \frac{\iint \iint\rho(t,a,\alpha,x,\xi) I (t,\alpha,\xi) S (t,a,x) \,\mathrm {d}{\alpha} \,\mathrm {d}{\xi} \,\mathrm {d}{a} \,\mathrm {d}{x}}{\iint ( \vartheta (t,a,x) I(t,a,x) + \eta(t,a,x) H (t,a,x) ) \,\mathrm {d}{a} \,\mathrm {d}{x}}. \end{aligned}$$

These latter simplified expressions still give useful information, since

$$\begin{aligned} &\tilde{\mathcal{R}}_{o} (t) < 1 \quad\implies \quad\frac{d}{d t} \biggl( \iint I (t,a,x) \,\mathrm {d}{a} \,\mathrm {d}{x} \biggr) < 0, \\ &\tilde{\mathcal{Q}}_{o} (t) < 1 \quad\implies\quad \frac{d}{d t} \biggl( \iint \bigl( I (t,a,x) + H (t,a,x) \bigr) \,\mathrm {d}{a} \,\mathrm {d}{x} \biggr) < 0. \end{aligned}$$

We remark that on relatively short time intervals (up to, say, a year or so), the difference between $\mathcal{R}_{0}$ and $\tilde{\mathcal{R}}_{0}$ (or between $\mathcal{Q}_{0}$ and $\tilde{\mathcal{Q}}_{0}$) is likely to be negligible.

As a further remark, note that under assumption (5), the instantaneous variation in the total population in the region $\mathcal{X}$ is

7 $$\begin{aligned} & \frac{\mathrm {d}{}}{\mathrm {d}{t}} \int_{\mathcal{X}} \int_{{\mathbb {R}}_{+}} (S + I + H + R) \,\mathrm {d}{a} \,\mathrm {d}{x} \\ &\quad= \int_{\mathcal{X}} S_{b} \,\mathrm {d}{x} \quad[ \text{newborn}] \\ &\qquad{} + \int_{\partial\mathcal{X}} \int_{{\mathbb {R}}_{+}} (v_{S} S + v_{I} I + v_{R} R) \cdot\nu \,\mathrm {d}{a} \,\mathrm {d}{\xi} \quad [\text{inflow/outflow}] \\ &\qquad{} - \int_{\mathcal{X}} \int_{{\mathbb {R}}_{+}} ( \mu_{S} S + \mu_{I} I + \mu_{H} H + \mu_{R} R ) \,\mathrm {d}{a} \,\mathrm {d}{x} \quad [ \text{deaths}], \end{aligned}$$

where $\nu= \nu(\xi)$ is the inward normal at *ξ* to $\partial\mathcal{X}$ and the boundary data $S_{b}$ measures newborns, see (4). The equality (7) clearly shows the role of the mortality rates $\mu_{S}, \mu_{I}, \mu_{H}, \mu_{R}$.

An obvious consequence of (1) is that, for the epidemic to arise, it is necessary that infective individuals are either present or enter the domain $\mathcal{X}$. Indeed, if $I_{o} (a,x) \equiv0$ and $I_{\partial} (t,a,\xi) \equiv0$, then the whole population remains forever untouched by the virus.

A key structural property of (1) is that the first two equations are independent of the latter two. Once *S* and *I* are known, the explicit forms of *H* and *R* are available through, for instance, a mixing of [13, Lemma 4.10] and [14, Lemma 3]. Formally, system (1) is a system of balance laws in several space dimensions. For these kind of partial differential equations, a general well posedness theory is still missing. However, the different equations are coupled through the source terms, similarly to the cases considered in [8, 15] where well posedness is obtained, as well as the stability with respect to the parameters defining the equation, see [16].

Different *costs* are related to the pandemic. First, the total number of deaths due to the disease on the time interval $[0,T]$, say $\mathcal{D} (T)$, is probably the most relevant one:

$$\begin{aligned} \mathcal{D} (T) = \int_{0}^{T} \int_{{\mathbb {R}}_{+}} \int_{ \mathcal{X}} \bigl(\mu_{I} (t,a,x) I (t,a,x) + \mu_{H} (t,a,x) H (t,a,x) \bigr) \,\mathrm {d}{x} \,\mathrm {d}{a} \, \mathrm {d}{t}. \end{aligned}$$

We do not enter here the issue of assigning the cause of the death to the virus in presence of other health problems.

On the other hand, we can also consider a more general cost comprising, for instance, also the expenses that the health system must sustain. Therefore, we refer to the cost functional

$$ \mathcal{C} (T) = \int_{0}^{T} \int_{{\mathbb {R}}_{+}} \int_{ \mathcal{X}} C \bigl(t,a,x, I (t,a,x), H (t,a,x) \bigr) \, \mathrm {d}{x} \,\mathrm {d}{a} \,\mathrm {d}{t}. $$

Above, the explicit dependence of *C* on $t,a,x$ may account for the peculiarities that different time periods, ages or regions may have.

The many policies or strategies that can be adopted to confine the infection enter *ρ* and the various parameters in (1). Besides, a quite natural choice is to use as control the function *κ*, since it describes the rate at which infective individuals are confined.

Before passing to simplified versions of (1), we note that generalizations and extensions are also possible. First, each population can be split into females and males, for instance. On long time intervals, the introduction of *growth functions*, accounting for the different aging of the different populations, might also be considered.

Hopefully, a particularly hot topic in the next future will be the strategy to adopt when a vaccine will be available. From the modeling point of view, this amounts to insert vaccination in the present model, following the framework in [17].

Finally we recall that, in system (1), recovered individuals cannot be infected again. The mostly unfortunate case where this assumption were false would amount to the introduction of further terms on the right hand sides.

### Simplified versions

While the model (1) looks quite general, in its use on a time scale of, say, a year or less, the terms $\partial_{a} S$, $\partial_{a} I$, $\partial_{a} H$ and $\partial_{a} R$, typically describing the aging of the population, can be neglected. Then, (1) reduces to the system of partial differential equations

8 $$ \textstyle\begin{cases} \partial_{t} S + \mathop {\operatorname {div}}_{x} (v_{S} S) + \mu_{S} S = - (\rho\otimes I) S, \\ \partial_{t} I + \mathop {\operatorname {div}}_{x} (v_{I} I) + \mu_{I} I = \hphantom{+ }(\rho\otimes I) S - \kappa I - \vartheta I, \\ \partial_{t} H + \mu_{H} H = + \kappa I - \eta H, \\ \partial_{t} R + \mathop {\operatorname {div}}_{x} (v_{R} R) + \mu_{R} R = + \vartheta I + \eta H, \end{cases} $$

where the age variable *a* can be considered as a parameter. Note that, as in the general case, the latter two equations can be explicitly solved, as soon as a solution to the system consisting of the former two equations is available.

If moreover we neglect the spatial velocity, i.e, we set $v_{S} = v_{I} = v_{R} = 0$, then system (8) becomes

9 $$ \textstyle\begin{cases} \dot{S} + \mu_{S} S = - (\rho\otimes I) S, \\ \dot{I} + \mu_{I} I = \hphantom{+ } (\rho\otimes I) S - \kappa I - \vartheta I, \\ \dot{H} + \mu_{H} H = + \kappa I - \eta H, \\ \dot{R} + \mu_{R} R = + \vartheta I + \eta H, \end{cases} $$

where the dot symbol stands, as usual, for the time derivative. In the latter system, also the space variable *x* plays the role of a parameter.

## The numerical algorithm

The numerical integration of the simplified model (9) amounts to the approximate solution of ordinary differential equations where age (*a*) and space (*x*) play the role of parameters. The integral coupling in the right hand side of the first two equations of (9) is computed by means of a quadrature formula at each time step. Then, it is added to the other terms in the right hand side of (9) and an approximate solution is obtained using the exact solution to the linear (or, more precisely, affine) ODE consisting of the left hand sides alone. This stratagem allows to comply with the global balance (7) of all the populations, while ensuring that the approximate solutions are non negative. For the sake of completeness, we specify that all meshes are fixed and uniform. The age boundary $a=0$ as well as the geographic boundary in the *x* variable need no specific treatment, since the convective term is here absent.

The integration of the PDE system (8) requires far more attention. Here, the convective terms are all approximated through the classical Lax–Friedrichs scheme [18, Sect. 12.5]. We use dimensional splitting [18, Sect. 19.5] to combine the movements in the 2 space dimensions. A further splitting is used to cope with the various source terms [18, Sect. 17.1]. The time step is chosen adaptively, with a CFL number [18, Sect. 4.4] of 0.95.

A technical issue is worth a specific remark. The presence of the non local term $\rho\otimes I$, see (2), requires the computation of an integral over all the spatial domain at each time step. The use of a suitably refined spatial mesh makes these computations quite demanding, in particular for what concerns computational time and, at a minor extent, memory requirements.

Numerical methods specifically devoted to conservation, or balance, laws with non local terms have been thoroughly developed in recent years. This development was often motivated by specific applications, ranging for instance from supply chains [19, 20], to the cutting of metal plates with laser beams [21, Sect. 4.2], to mixed hyperbolic–parabolic predator–prey systems [22].

## The role of quarantine

We now show that the presented model, though in the simplified form (9), does capture the relevance of the quarantine. Throughout, we use the parameters computed in the Appendix, in particular we refer to the mortalities in Table 1 therein. We integrate three instances of (9) differing exclusively in the values attained by *κ*. Recall that this parameter quantifies the speed at which infective individuals are confined to quarantine.

**Table 1:** Mortalities

Age class	[0,40[	[40,60[	[60,80[	[80,+∞[	See
Total Deaths in 2018	7893	38,896	182,282	404,062	[24]
Residents in 2018	24,421,783	18,640,004	13,215,186	4,207,000	[23]
$\mu_{S} (a) = \mu_{R} (a) =$	8.85e-7	5.72e-6	3.78e-5	2.63e-4	(18)
Covid-19 deaths (up to 15.03):	4	55	746	892	[25]
Covid-19 deaths (up to 22.03):	12	209	2309	2488	[26]
Covid-19 cases (up to 15.03):	2953	7729	9561	4636	[25]
Covid-19 cases (up to 22.03):	6891	18,547	21,743	10,514	[26]
$\mu_{H} (a) = \mu_{I} (a) =$	2.90e-4	2.03e-3	1.83e-2	3.87e-2	(19)

In the first case, we set $\kappa\equiv0$, then we use *κ* as defined in (20), in the third integration we use 10 times the value of *κ* in (20) and in the latter integration we use 20 times the *κ* in (20).

We detail the choices of the initial datum (4):

10 $$\begin{aligned} \begin{aligned} S_{o} (a,x) = {}& [ 3.125 \chi _{\strut [0, 40[} (a) + 4.688 \chi _{\strut [40,60[} (a) + 3.125 \chi _{\strut [60,80[} (a) \\ &{}+ 1.563 \chi _{\strut [80, +\infty[} (a) ] \chi _{\strut [-40, 40]\times[-40, 40]} (x); \\ I_{o} (a,x) ={}& 8 \chi _{\strut \{x \colon{ \vert x_{1}+20 \vert } + { \vert x_{2}-20 \vert } < 0.5 \}} (x) + 32 \chi _{\strut \{x \colon{ \vert x_{1}-3 \vert } + { \vert x_{2}+2 \vert } < 0.25 \}} (x) \\ &{}+ 80 \chi _{\strut \{x \colon{ \vert x_{1}-38 \vert } + { \vert x_{2}-36 \vert } < 0.4 \}} (x) + 4 \chi _{\strut \{x \colon{ \vert x_{1}+10 \vert } + { \vert x_{2}+20 \vert } < 0.3 \}} (x) \\ &{}+ 28 \chi _{\strut \{x \colon{ \vert x_{1}-28 \vert } + { \vert x_{2}+9 \vert } < 0.6 \}} (x); \\ H_{o} (a,x) ={}& 0; \\ R_{o} (a,x) ={}& 0. \end{aligned} \end{aligned}$$

The dynamics of infection is described by the function *ρ* which we here select as follows:

$$ \rho(t,a,\alpha,x,\xi) = 0.005 \chi _{\strut \bigl\{ (x,\xi) \colon{ \Vert x-\xi \Vert } < 1.5 \bigr\} } (x,\xi). $$

This choice amounts to allow infection to pass from infective to susceptibles only provided that individuals are less than 1.5 apart. The transmission of the disease takes place independently of the age and of the absolute positions, the only constraint being the vicinity of infective and susceptibles. We also choose that the transmission of the disease is independent of time.

**Figure 2:**
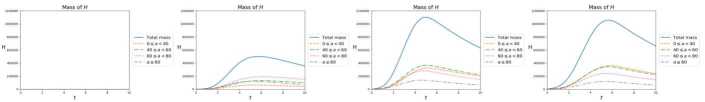
Total number of isolated individuals as a function of time, according to (9), in the four cases, from left to right: $\kappa= 0$, *κ* as in (20), 10*κ* and 20*κ*. Remarkably, in the latter case the peak of the map $t \to\iint H \,\mathrm {d}{a} \,\mathrm {d}{x}$ is lower than on the preceding case. Indeed, a high value of *κ* reduces the number of infectives and, as a consequence, may also reduce the total number of individuals in quarantine

**Figure 3:**
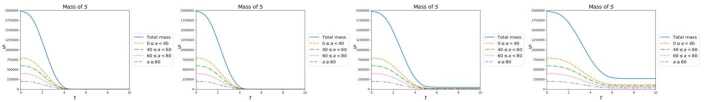
Total number of susceptible individuals as a function of time, according to (9), in the four cases, from left to right: $\kappa= 0$, *κ* as in (20), 10*κ* and 20*κ*. The increase in the infectives’ isolation speed slightly lengthen the time necessary for susceptibles to be infected

**Figure 4:**
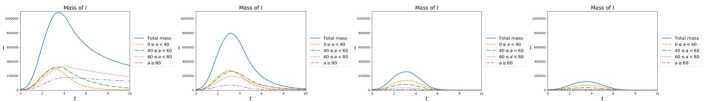
Total number of infective individuals as a function of time, according to (9), in the four cases, from left to right: $\kappa= 0$, *κ* as in (20), 10*κ* and 20*κ*. It is evident that quarantine sharply reduces the amount of infective individuals

**Figure 5:**
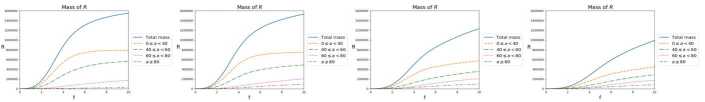
Total number of recovered individuals as a function of time, according to (9), in the four cases, from left to right: $\kappa= 0$, *κ* as in (20), 10*κ* and 20*κ*. The increase in the quarantined individuals leads to a decrease of the infected ones and, hence, also of those that recover

**Figure 6:**
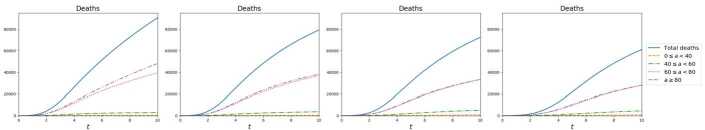
Total number of deaths due to the pandemic as a function of time, according to (9), in the four cases, from left to right: $\kappa= 0$, *κ* as in (20), 10*κ* and 20*κ*. Here, the effect of quarantine is evident, sharply reducing the death toll. Note also the slightly different effects on the different age classes

The effect of quarantine is well captured even by the simplified model (9). When *κ* is 0, no quarantine occurs and the virus spreads the fastest, see Fig. 2. Higher values of *κ* mean that more individuals are quarantined/hospitalized, slowing down the spreading of the virus. As *κ* increases, *S* individuals take more time to get infected, see Fig. 3. Clearly, with lower values of *κ*, the disease spreads more rapidly, so that the number of infectives is far higher, see Fig. 4, and less individuals recover, see Fig. 5. Moreover the total number of deaths decreases as *κ* increases, see Fig. 6. The present model, being age structured, accounts also for age differences in the death toll due to different quarantine levels.

Note the counter-intuitive effect according to which the highest value of *κ* does not correspond the highest peak in the map $t \to\iint H (t, a, x) \,\mathrm {d}{a} \,\mathrm {d}{x}$. Indeed, higher values of *κ* reduce the total number of infected people which, as a consequence, may well lead to a *reduction* in the number of isolated individuals, see Fig. 2.

Note that higher values of *κ* not only lead to lower values of $t \to\iint I (t,a,x) \,\mathrm {d}{a} \,\mathrm {d}{x}$, but also move the peak of this function to the right. From the practical point of view, this is likely to correspond to a lighter exploitation of intensive care units, a key aspect from the public health point of view.

**Figure 7:**
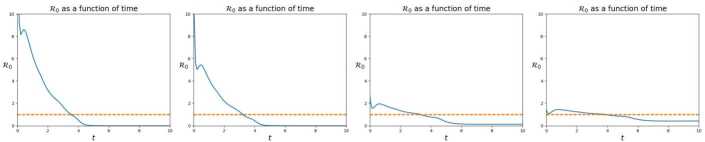
Value of the $\mathcal{R}_{0}$ index, as defined in (6), as a function of time, according to (9), in the four cases, from left to right: $\kappa= 0$, *κ* as in (20), 10*κ* and 20*κ*. The effect of quarantine is evident: increasing values of *κ* result in longer periods where $\mathcal{R}_{0}$ exceeds one, but with lower values of the index. This typically results in a more bearable stress on the health system

This *“slowing”* effect is evident also in Fig. 7: lower values of *κ* result in a shorter time interval where $\mathcal{R}_{0}$ exceeds 1. However, the values attained by this index may cause an excessive stress on intensive care units. On the contrary, higher values of *κ* lead to a longer period where $\mathcal{R}_{0}$ exceeds 1, but with values that suggest a minor stress on the health system.

## Residential care homes

A recurrent problem in several countries has been the propagation of Covid-19 in care homes. Here, we simulate this phenomenon, showing that the presence of a less controlled area, though containing only one age segment, not only directly suffers from the pandemic, but may well accelerate the virus propagation in the care homes’ neighborhoods.

To this aim, we now integrate (9) with the parameters, in particular the mortalities, detailed in the Appendix and with the following initial datum:

11 $$\begin{aligned} \begin{aligned} S_{o} (a,x) = {}& [ 3.13 \chi _{\strut [0, 40[} (a) + 4.69 \chi _{\strut [40,60[} (a) + 3.13 \chi _{\strut [60,80[} (a) \\ &{}+ 3.13 \chi _{\strut [80, +\infty[} (a) ] \chi _{\strut [-20, 20]\times[-20, 20]} (x) \chi _{\strut {\mathbb {R}}^{2} \setminus(C_{1}\cup C_{2})} (x) \\ &{}+ 1.56 \chi _{\strut [80, +\infty[} (a) ( \chi _{\strut C_{1}} (x) + \chi _{\strut C_{2}} (x) ); \\ I_{o} (a,x) ={}& 20 \chi _{\strut [40, 80[} (a) \chi _{\strut [0,20] \times[35, 40]} (x); \\ H_{o} (a,x) ={}& 0; \\ R_{o} (a,x) ={}& 0, \end{aligned} \end{aligned}$$

where the two care homes $C_{1}$ and $C_{2}$ are located at

12 $$ C_{1} = [-10, 10] \times[10, 20]\quad \text{and}\quad C_{2} = [-35, -25] \times[-30, -20]. $$

In these regions, only one age class, namely the oldest one, is present and, mostly, less protective measures are adopted. We describe this underestimation of the dangers related to the virus through the function *ρ*:

13 $$ \rho(t, a, \alpha, x, \xi) = \bigl( 8\times10^{-5} + 2.5 \times10^{-3} \chi _{\strut C_{1} \cup C_{2}} (x) \bigr) \chi _{\strut \bigl\{ (x,\xi) \colon{ \Vert x-\xi \Vert } < 5 \bigr\} } (x, \xi). $$

Note that in $C_{1}$ and $C_{2}$, *ρ* is about 30 times larger than outside these regions. This choice accounts for the easiness with which, tragically, contagion diffused in some Care Homes.

**Figure 8:**
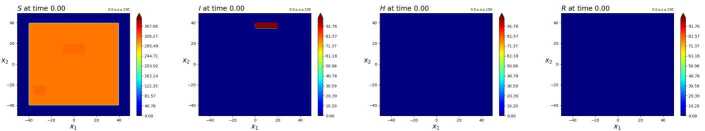
Contour plots of the initial datum as a function of the space variable for the *“Care Homes”* integration. Note that the total distribution of the *S* population is approximately uniform, while a small groups of infected individuals are present in the top part of the graph. Neither *H* nor *R* individuals are now present

**Figure 9:**
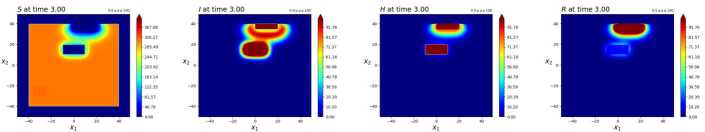
Contour plots of the solution to the *“Care Homes”* integration at time $t = 3$. Note the fast spreading of the disease in $C_{1}$ as defined in (12). From there, the virus spreads even faster

**Figure 10:**
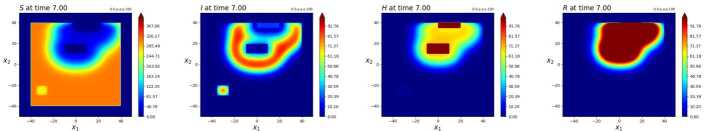
Contour plots of the solution to the *“Care Homes”* integration at time $t = 7$. Note that the care house $C_{2}$, as defined in (12), is reached by the virus through a very small amount of *I* individuals, so small that it is not highlighted with the current scale. Indeed, contrary to the impression suggested by these figures, the model (9) does not allow for any propagation at a distance greater than $\delta= 5$, as specified in (13)

**Figure 11:**
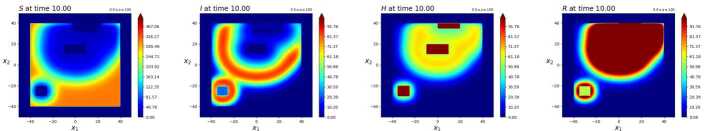
Contour plots of the solution to the *“Care Homes”* integration at time $t = 10$. The front of the pandemic clearly spreads from the top right towards the bottom left of the domain and now the care house $C_{2}$, as defined in (12), acts as an epidemic outbreak, opening a second front and accelerating the pandemic in the lower left part of the domain

**Figure 12:**
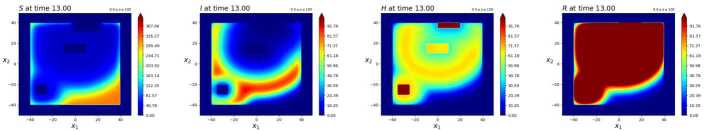
Contour plots of the solution to the *“Care Homes”* integration at time $t = 13$. The front of the pandemic for the *I* population spreads from the care house $C_{2}$, as defined in (12)

At time $t=0$, the *S* population is (approximately) uniformly distributed in $[-40,40] \times[-40,40]$. In $C_{1}$ and in $C_{2}$ only members of the oldest age group (i.e., $a > 80$) are present, see Fig. 8. Quickly, at time $t=3$, the virus reaches the first care home $C_{1}$, see Fig. 9. As a consequence, the pandemic accelerates and, at time $t=7$, also $C_{2}$ is widely infected, see Fig. 10. This further accelerates the spreading, with $C_{2}$ clearly acting as a further source of infection, see Fig. 11. At time $t=10$, the two fronts of the pandemic propagation are evident: the first one due to the initial presence of infected individuals, the second one emanating from $C_{2}$. Finally the situation at time $t = 13$ is plotted in Fig. 12.

**Figure 13:**
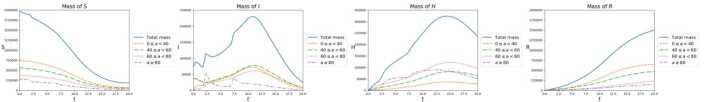
Total amount of individuals of the four populations over all the domain and all age classes, as a function of time, with reference to the *“Care Homes”* integration. Remarkably, the initial tendency of the *I* population is towards a reduction but, as soon as the virus reaches $C_{1}$, at about $t=2$, this tendency is reverted and the pandemic accelerates

In Fig. 13, we see the total amounts of individuals of all ages and over all the domain. Note, in particular, that the initial trend of the *I* population is towards a *decrease*. Nevertheless, the outbreak of the pandemic in the first care home $C_{1}$ is able to invert this trend and the total number of infected individuals starts to grow.

**Figure 14:**
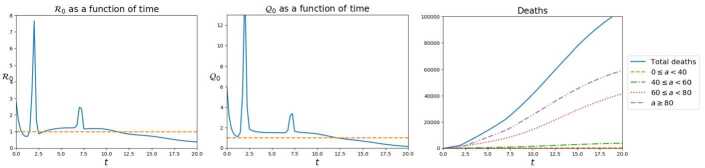
$\mathcal{R}_{0}$, left, and $\mathcal{Q}_{0}$, middle, indexes as a function of time in the simulation of the *“Care Homes”* case. The epidemic outbreaks in $C_{1}$ and $C_{2}$ are clearly visible. Right, the total number of deaths due to the pandemic, as a function of time, also separated in the 4 age classes. As expected, the oldest fraction of the population pays the highest toll

Finally, remark that although the two care homes are rather small with respect to the whole domain, the spreading of the virus in $C_{1}$ and in $C_{2}$ is very clearly caught by the indexes $\mathcal{R}_{0}$ and $\mathcal{Q}_{0}$ defined in (6), see Fig. 14. Indeed, the two care homes actually accelerate the contagion in the neighboring areas. A further expected consequence is the soaring number of casualties in the highest age classes, confirmed in Fig. 14.

As a general remark, we stress that this integration well captures the dangerous consequences that the pandemic may have on localized areas where the disease propagation is not sufficiently hindered. Indeed, the positions of the two “Care homes” are undistinguishable when looking at the population density distribution at time $t=0$, see Fig. 8. Nevertheless, as soon as infection reaches these regions, it spreads therein quite quickly, see in particular the two rightmost diagrams in Fig. 9, where a hole in the *S* density and a sharp increase in the *I* density clearly correspond to the propagation of the disease in a care home. In the subsequent Fig. 10, this phenomenon is even more evident: the second care home is fully infected without any apparent contagious wave reaching it. Clearly, once a care home is infected, it acts as a further source of infection for the neighboring regions. However, this effect is better accounted for when introducing also movements in space.

## The effects of spatial movements

The spatial structure present in (1) allows to describe the relevant effects that the movements of individuals may have on the spreading of the virus.

We imagine an urban area with commuters focusing in a fictitious city center every morning and going back home in the evening. Slightly out of the commuting area, a group of infected individuals initiate the spreading of the virus. The pandemic turns out to be dramatically enhanced by the commuters’ movements.

More precisely, we use model (1) in the form (8), with no age structure, on the domain $[-2.1, 2.1] \times[-2.1, 2.1]$, with an initial datum corresponding to a uniform distribution of *S* individuals, with density $206 \frac{1}{\text{Km}^{2}}$ as detailed in the Appendix, in $[-2, 2] \times[-2, 2]$ and a 20 times smaller density of infective ones in a corner:

14 $$ \textstyle\begin{cases} S_{o} (x) = 206 \chi _{\strut [-2, 2] \times[-2, 2]} (x), \qquad H_{o} (x) = 0, \\ I_{o} (x) = 10.3 \chi _{\strut [-1.9, -1.5] \times[-1.9, -1.5]} (x), \qquad R_{o} (x) = 0. \end{cases} $$

The movement of the commuters is imitated through the vector field

15 $$ v (t,x) = \textstyle\begin{cases} -2 ( { \Vert x \Vert }^{2} - (7/4)^{2} ) x \chi _{\strut { \Vert x \Vert } \leq7/4}& \text{between $06{{:}}00$ and $09{:}00$}, \\ 0 &\text{between $09{:}00$ and $17{:}00$}, \\ 2 ( { \Vert x \Vert }^{2} - (7/4)^{2} ) x \chi _{\strut { \Vert x \Vert } \leq7/4} &\text{between $17{:}00$ and $20{:}00$}, \\ 0 &\text{between $20{:}00$ and $06{:}00$}, \end{cases} $$

which has a period of 1 day.

The function *ρ* depends only on the space variables *x* and *ξ* and is chosen as

16 $$ \rho(t, a, \alpha,x, \xi) = \chi _{\strut \bigl\{ (x,\xi) \colon{ \Vert x-\xi \Vert } < 0.05 \bigr\} } (x,\xi). $$

The other parameters are age–averages of the quantities chosen in the Appendix, namely:

17 $$ \begin{aligned} &\mu_{S} = \mu_{R} = 2.87 \times10^{-5} \frac{1}{\text{day}};\qquad\eta = 0.115 \frac{1}{\text{day}}; \\ &\kappa= 0.320 \frac{1}{\text{day}};\\ &\mu_{I} = \mu_{H} = 1.44 \times10^{-2} \frac{1}{\text{day}};\qquad \vartheta = 0.420 \frac{1}{\text{day}}. \end{aligned} $$

**Figure 15:**
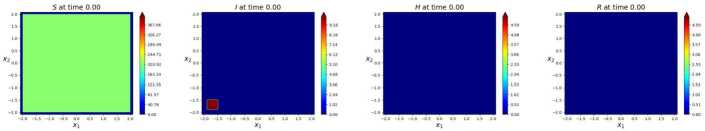
Initial datum assigned to (8) in the two cases with and without commuters. The commuting area is the circle centered at the origin with radius 1.75. From left to right, the *S*, *I*, *H* and *R* populations

As a comparison, we consider the same situation but with the vector field (15) replaced by 0. The initial datum is the same in the two cases and is depicted in Fig. 15. Note that the initial distribution of infected individuals is out of the residential area of the commuters. Therefore, as long as the virus does not reach the circle centered at $(0,0)$ with radius 1.75, that is for the first 3 days, the two evolutions roughly coincide.

**Figure 16:**
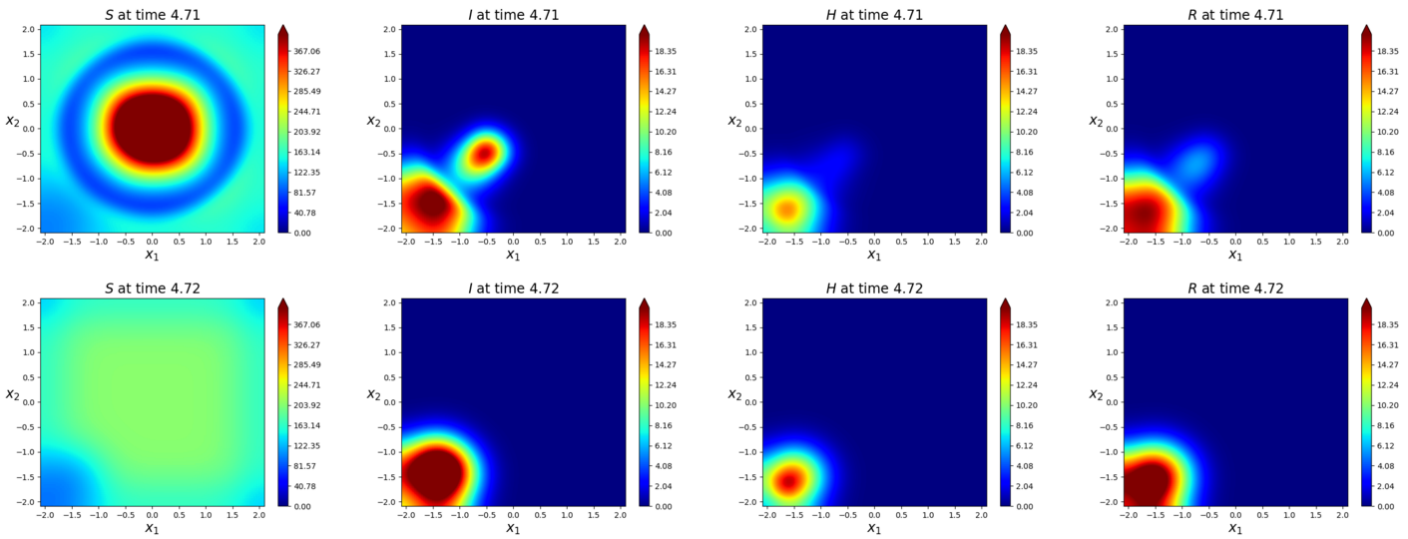
Contour plots of the solution to (8) at time, above, $t=4.71$ and, below, $t = 4.72$, roughly corresponding to 5 p.m. of the 4^th^ day. Above, the case with the commuters’ movement (15), below the case with no movement. Apart from the spreading of the virus in the bottom left corner, the *S* distribution in the line above is symmetric w.r.t. the origin, while on the line below it is uniform. Here, differences in the virus spreading due to daily travelers are becoming evident

**Figure 17:**
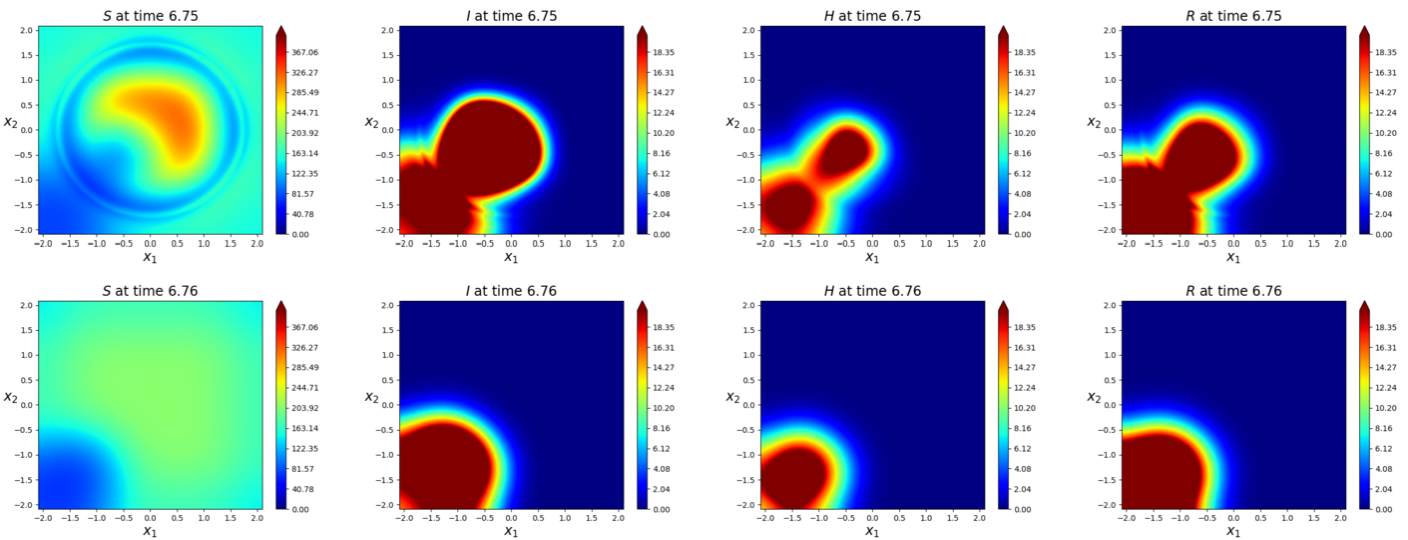
Solution to (8) at time, above, $t=6.75$ and, below, $t = 6.76$, roughly corresponding to 6 p.m. of the 4^th^ day. Recall that the vector field (15), used in the integration above, is 1 day periodic. Above, the combined effect of 6 days of commuting clearly results in a higher level of infection

Each morning, the focusing of all individuals not in quarantine in the center increases the total individuals’ density therein. Thus, during working hours, i.e., between 09:00 and 17:00, the higher density in the center eases the virus propagation. In Fig. 16, we see the first effects of the daily travelers: they clearly speed up the virus propagation. At about 5 p.m. of the 4^th^ day, a group of infected individual is present in the city center. Later, in the evening, infection is propagated back near to the homes of the commuters. The periodicity in these daily travels boosts virus propagation, see Fig. 17, where the infected area in the commuters’ case is considerably larger than that with no movement.

**Figure 18:**
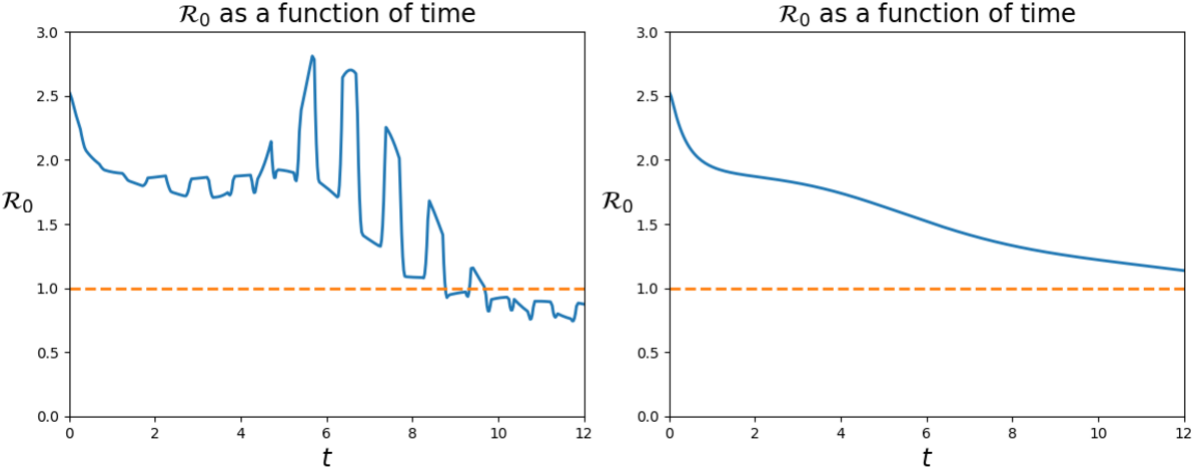
Value of the $\mathcal{R}_{o} (t)$ index corresponding to the solution to (8) on a 12 days time interval. The commuters’ movements clearly boost the spreading of the virus

The two graphs of the $\mathcal{R}_{o}$ index confirm the previous remarks. During working hours, when the *S* and *I* individuals are concentrated in the center, the diffusion of the pandemic is evidently amplified, see Fig. 18. Towards the end of the time interval, the value of $\mathcal{R}_{o}$ falls below 1 since most of the population is, or was, infected.

**Figure 19:**
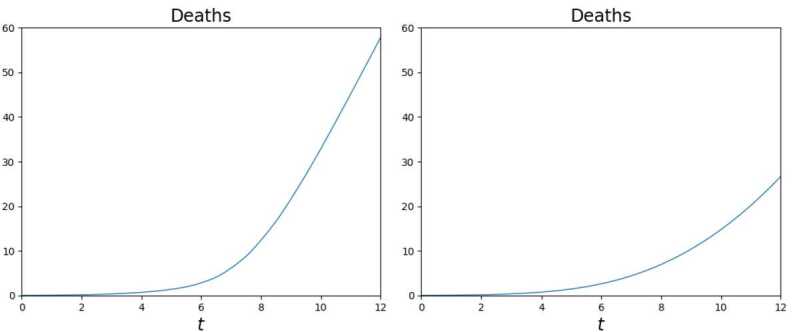
Numbers of deaths due to the virus, as a function of time, according to the solution to (8) in 12 days. The daily workers’ movements double the number of casualties

As a *compendium* of the two different evolutions, the graph of the total numbers of deaths in Fig. 19 is dramatically clear. The movement of commuters doubles the number of casualties.

Finally, we remark that different choices of the vector field (15) can easily increase the difference in the death toll. Indeed, assume, for instance, a movement allowing the infective individuals to wander all around the region under consideration. In such a configuration, the movement dramatically amplifies the infectivity of the virus. The setting considered above was chosen to be reasonably realistic and, nevertheless, still shows the relevance of individuals’ movements in the spreading of the virus.

## Conclusions

The key novelties in the model presented above are the non local integral term representing the transmission of infection and the explicit consideration of a segment of the population confined to quarantine. The resulting model consists of an initial boundary value problem for a system of partial differential equations where the independent variables are time, age and the space coordinates.

## Electronic Supplementary Material

Below are the links to the electronic supplementary material.

Integration of (9)–(10) with $\kappa=0$, *κ* as in (20) and 10 times *κ* as in (20). Integration of (9) with initial datum as in (10) and the parameters described above. The three integrations differ in the choice of *κ*, which is assigned the value 0, the values in (20) and 10 times this latter value. The file names are:

**Table:** 

kappa0.avi	corresponding to *κ* = 0;
kappa1.avi	corresponding to *κ* as in (20)
kappa2.avi	corresponding to 10 times the value of *κ* in (20).
kappa3.avi	corresponding to 20 times the value of *κ* in (20).

(ZIP 2.2 MB)

Care Homes Integration. Integration of (9) with initial datum as in (11)–(13). The file name is CareHomes.avi. (ZIP 498 kB)

The Effects of Spatial Movements. Integration of (8) with initial datum as in (14). The case with spatial movement is in file MovingYes.avi, while the case with no movement is in MovingNo.avi. (ZIP 2.5 MB)

## Appendix: Parameters’ choices

We detail here the procedures adopted to select the values of the parameters used.

In the integrations, we are inspired by average Italian data, scaled to the square $[-50, 50] \times[-50, 50]$, with a total population of about 2 millions inhabitants, approximating the average Italian population density of $206 \frac{1}{\text{Km}^{2}}$.

As common experience shows, age plays a role in the evolution and consequences of the Covid-19 infection. Therefore, we distinguish 4 age classes (in years): $[0, 40 [$, $[40, 60 [$, $[60, 80 [$ and $[80, +\infty [$. We recall that the total population is distributed among these age classes, following [23], with the coefficients:

$$\begin{aligned} \textstyle\begin{array}{l@{\quad}|@{\quad}l@{\quad}|@{\quad}l@{\quad}|@{\quad}l@{\quad}|@{\quad}l} \text{age class} & [0, 40 [ & [40, 60 [ & [60, 80 [ & [80, +\infty [ \\ \hline\text{density distribution} & 0.404 & 0.308 & 0.218 & 0.0700 \end{array}\displaystyle \end{aligned}$$

The estimation of all quantities related to the *I* population is intrinsically quite difficult. These individuals are infective but not isolated, therefore they may be unknown to any agency, as they may well be unaware of their status. It is generally believed that their number exceeds the official number of positive tests, but very little seems to be known about the other parameters specific to the *I* population, such as mortality, for instance.

*Mortalities*. Remark that here our aim is to capture qualitative features or compare different strategies to cope with the pandemic, rather than obtain quantitatively correct forecasts. Therefore, we are more interested in the *ratios* among the different mortalities, rather than in their absolute values. Dimensionally, mortalities are measured by $\frac{1}{\text{day}}$.

The mortalities $\mu_{S}$ and $\mu_{R}$ are computed, in each age interval, so that

18 $$ (365~\text{day}) \mu \text{[individuals in a class]} = \text{[deaths in that class in 2018]}, $$

while to compute the values of $\mu_{H}$ we used

19 $$ (7~\text{day}) \mu \begin{bmatrix} \text{infected individuals} \\ \text{in an age class} \\ \text{between 15.03 and 22.03} \end{bmatrix} = \begin{bmatrix} \text{deaths in that age class} \\ \text{between 15.03 and 22.03} \end{bmatrix} . $$

In the above computations leading to the estimates of the mortalities, we assumed that all those who are aware of being infective belong to the *H* population, meaning that they do not infect anyone.

Here, we set $\mu_{I} = \mu_{H}$. Indeed, the *I* population both comprises asymptomatic individuals that hardly realize being infected, as well as those who are not taken care of. In the average, we arbitrarily assume that their mortality is as big as that of the isolated individuals.

*Transitions between populations*. The parameter $\kappa= \kappa(t, a, x)$ measures the rate at which infective individuals are *“blocked”*, i.e., hospitalized or set to quarantine. Due to the above recalled nature of the *I* population, we estimate *κ* as being generally larger than $\mu_{I}$. Indeed, we expect that infective individuals usually become known and, hence, isolated well before their conditions get too bad. Moreover, we expect that *κ* is bigger at higher ages, since the presence of infective individuals that are not aware of their status (and, hence, are not isolated) might be larger at lower ages. Thus, we choose *κ* as being only age dependent and, more precisely, we set^1^

20 $$ \kappa(t,a,x) = 0.1 \chi _{\strut [0, 40[} (a) + 0.2 \chi _{\strut [40, 60[} (a) + 0.4 \chi _{\strut [60, 80[} (a) + 0.8 \chi _{\strut [80, +\infty[} (a). $$

Recall that *κ* plays a key role in the control of the epidemic and a paragraph below is devoted to show its relevance.

The parameter *ϑ* is the speed at which infective individuals recover. It is realistic to assume that this happens at a rate faster than the death rate and, what is more relevant, faster for younger individuals. Thus, neglecting the dependence on time and space, we set

$$\begin{aligned} \vartheta (t,a,x) = 0.8 \chi _{\strut [0, 40[} (a) + 0.4 \chi _{\strut [40, 60[} (a) + 0.08 \chi _{\strut [60, 80[} (a) + 0.02 \chi _{\strut [80, +\infty[} (a). \end{aligned}$$

Finally, *η* is the speed at which hospitalized individuals recover. We get from [27] the total number of individuals that recovered on March 23rd, namely 7342 out of a total *H* population on that day of $63\text{,}927$, so that we set

$$\begin{aligned} \eta(t,a,x) = 0.115 \frac{1}{\text{day}}. \end{aligned}$$

Note that the parameter *η* correctly turns out to be an average of the values attained by *ϑ*. The use of an age or, possibly, also time and space dependent *η* is well within the capabilities of Model (1).
